# Cost-effectiveness of school-based interventions for well-being and mental health literacy of pupils in year 9 in England: AWARE cluster randomised controlled trial

**DOI:** 10.1192/bjo.2026.11015

**Published:** 2026-06-08

**Authors:** Kyann Zhang, Derek King, Jan Boehnke, Jessica Deighton, Abigail Thompson, Emma Thornton, Christopher Knowles, Daniel Hayes, Paul Stallard, Joao Santos, Emma Ashworth, Sara E. Evans-Lacko

**Affiliations:** Care Policy and Evaluation Centre, London School of Economics and Political Science, London, UK; School of Health Sciences, University of Dundee, UK; Evidence Based Practice Unit, University College London and Anna Freud Centre, UK; Manchester Institute of Education, The University of Manchester, UK; Department of Behavioural Science and Health, University College London, UK; Department of Health, University of Bath, UK; School of Psychology, Liverpool John Moores University, UK

**Keywords:** Mental health, health economics, cost-effectiveness analysis, education, quality of life

## Abstract

**Background:**

AWARE (Approaches for Wellbeing and Mental Health Literacy: Research in Education) is a three-arm, parallel-group, cluster randomised controlled trial. It assessed the effectiveness of two interventions – the Youth Aware of Mental Health (YAM) programme and Mental Health and High School Curriculum Guide – in addressing emotional well-being, compared with usual practice, among year 9 students in England.

**Aims:**

To evaluate the cost-effectiveness of YAM and The Guide to inform policy decisions regarding the implementation of these mental health interventions in schools.

**Method:**

Cost-effectiveness was assessed using self-reported information from participants in the trial at baseline and two follow-ups – at 3–6 months after the start of intervention, and at 9–12 months post-intervention. Quality of life was measured with the Child Health Utility Index. Intervention delivery costs were calculated with data provided by the delivery teams. Service use costs were calculated with a short version of the Client Service Receipt Inventory, with unit costs obtained from publicly available sources.

**Results:**

For both interventions, difference in outcomes (change in quality-adjusted life-years over time) between the intervention group and control group were close to zero, with the largest change being an improvement of 0.0055 quality-adjusted life-years at the second follow-up for students in schools randomised to YAM. Changes in costs were likewise small.

**Conclusions:**

At first follow-up, neither intervention is likely to be considered cost-effective. At second follow-up, YAM has a high probability of being considered cost-effective, with an incremental cost-effectiveness ratio of around £23 000 per unit of improvement in the quality-of-life measure, which falls within the threshold (£20 000 to £30 000) as used by the National Institute for Health and Care Excellence.

The cost of mental health problems in England has been estimated to exceed £100 billion.^1^ It is suggested that much of this cost could be mitigated by investments in early prevention interventions – notably, initiatives during key developmental stages of childhood and early adolescence. Schools have been identified as a universal point of access for young people in key developmental stages, which makes them effective platforms for early prevention and mental health promotion.^2^ Previous studies have found some evidence for the effectiveness of school-based programmes in promoting mental health;^3^ however, it has also been noted that such interventions have not been investigated in the UK to the same extent as in other countries, such as the USA.^4^ Further, evidence on the cost-effectiveness of such interventions is limited, with few trials including an economic component in the analyses. A review in 2020^5^ found only nine studies (since 2013) of universal mental health interventions for children and adolescents that included an economic evaluation – only three of which took place in the UK. The review highlighted a number of challenges in addressing the cost-effectiveness of interventions, including the lack of cost-related information available. With consideration for the increasing demands on health and social care resources, it is important that cost-effectiveness forms part of the decision-making process with regard to intervention recommendations.

## Study objectives

This study aims to evaluate the cost-effectiveness of two school-based interventions, funded by the Department for Education, designed to improve mental health literacy and emotional well-being in year 9 students in England: (a) the Youth Aware of Mental Health (YAM) programme, a universal intervention to improve awareness and promote mental health;^6^ and (b) the Mental Health and High School Curriculum Guide (The Guide),^7^ which aims to increase awareness of mental disorders and their treatments, as well as increasing understanding of how to achieve and maintain mental health, reduce stigma and improve help-seeking efficacy. A description of these programmes are available from the Department for Education.^8^

The key objectives of this evaluation are: (a) to assess the cost-effectiveness of each of these interventions against usual school provision in terms of improving students’ health-related quality-of-life (HRQoL), as measured by quality-adjusted life-years (QALYs); (b) to assess the cost-effectiveness of YAM relative to usual school provision, with respect to changes in depressive symptoms; and (c) to assess the cost-effectiveness of The Guide relative to usual school provision, with respect to changes in help-seeking behaviour.

## Method

This health economic analysis was conducted alongside a three-arm, parallel-group, cluster randomised controlled trial (trial registration number: ISRCTN17631228) designed to evaluate the cost-effectiveness of the YAM programme, The Guide, and usual school provision for year 9 students in England. Recruitment took place between March 2018 and July 2019. Data were collected at three time points: at baseline, at 3–6 months after the start of the intervention and at 9–12 months following the end of intervention (as detailed in the pre-registered statistical analysis plan^9^). All questionnaires were completed online.

The target population was year 9 students (aged 13–14 years) from 144 secondary schools in England, totalling approximately 8600 participants. Parents/guardians of the pupils in these classes were sent letters informing them of the study. For outcome (effectiveness) data, opt-out consent was used when sending letters to parents/guardians of pupils who were selected by schools to take part. Parents/carers who wished to opt out their children were asked to return an opt-out form to the research team. Pupils whose parents had not opted out of the evaluation were given the option to assent to take part through reading an online information sheet and ticking an online assent form. Any young people that did not provide assent at the baseline survey did not take part in the trial. Data were collected via self-reported questionnaires from participants (staff and students). Financial information relating to intervention delivery, including training, were collected using financial surveys completed by school staff. Participant characteristics are presented in Table 1.

**Table 1:** Participant characteristics at baseline (*N* = 19 121)

Characteristic	YAM (*n* = 6306)	The Guide (*n* = 6398)	Usual practice (*n* = 6417)
Male, *n* (%)	2893 (46)	2975 (46)	3375 (53)
City, *n* (%)	3063 (49)	2942 (46)	3029 (47)
Free school meals eligibility, *n*			
Low	2057	2201	2204
Medium	2088	2207	2141
High	2161	1990	2072

YAM, Youth Aware of Mental Health programme; The Guide, Mental Health and High School Curriculum Guide.

Details of the study are reported in Deighton et al.^10^ A full description of the trial design is presented in the trial protocol,^11^ and the analyses plan was registered on Open Science Framework.^9^ Health economic analysis was conducted in line the Consolidated Health Economic Evaluation Reporting Standards reporting guideline.^12^ The analysis was conducted from a public health and social care perspective, including the costs of service use and intervention delivery.

The authors assert that all procedures contributing to this work comply with the ethical standards of the relevant national and institutional committees on human experimentation and with the Helsinki Declaration of 1975, as revised in 2013. All procedures involving human participants were approved by the University College London Research Ethics Committee (approval numbers 6735/009 and 6735/014).

### Outcome measures

In addition to the primary outcomes reported in the main analysis – the Short Mood and Feelings Questionnaire for YAM,^13^ and the General Help-Seeking Questionnaire for The Guide,^14^ – this health economic analysis analysed the self-reported quality-of-life scores, using the Child Health Utility 9D (CHU9D) Index. QALYs were calculated using preference weights estimated by Stevens.^15^ One QALY represents a year at full health. All models were adjusted for baseline costs and outcome measures. All instruments used have been validated for the age group in this study. Covariate measures included socioeconomic status, measured using level (low, medium, high) of free school meal eligibility in each participating school; school location and setting (rural versus urban), and any previous implementation of universal mental health programmes before involvement in this trial.

### Costs

#### Service use costs

Service use data were collected using a short form of the Client Service Receipt Inventory (CSRI),^16^ which were completed by students at baseline and at each of the two follow-ups. The CSRI collected information on the use of school, health (including hospital) and social services by students because of concerns about their thoughts, feelings or behaviour. To better facilitate recall, the questions were framed in terms of frequency since ‘the beginning of the school year’, with a multiple-choice style response. For example, the student would be asked ‘Since the beginning of the school year, have you seen [a teacher] because of worries about your thoughts, feelings or behaviour?’, with the options of responding **‘**once per day’, **‘**once per week’, and so on. See the Technical Report^17^ for the full questionnaire. Given that both follow-up surveys were conducted over a period of 3 months, the amount of time that passed between the ‘start of the school year’ and the follow-up was not a set length of time. To ensure consistency between student responses, and allow comparability between pre- and post-intervention periods, these frequencies were converted to annual estimates.

Cost of service use was calculated by combining service use with relevant unit costs. Unit costs were sourced from the latest version of publicly available publications, primarily from NHS Reference Costs,^18^ and the Unit Costs of Health and Social Care.^19^ Further information on hourly rates for school staff and social workers were obtained from The Teacher’s Union and the Office of National Statistics. See Table 2 for details. Although the CSRI included a question on the use of prescription medication, information on specific type or duration of prescribed medications was not collected in the interest of privacy. Students instead were asked to indicate whether they have been prescribed medication to address emotional concerns during the study period. Where students reported the affirmative, we include a one-off cost of issuing the prescription.

**Table 2:** Service unit costs

Services	Unit	Cost (£)	Source
School services			
Teacher	Annual	35 843.00	The Teacher’s Union
Teaching assistant	Annual	18 500.00	National Career Service
School nurse	Annual	33 124.00	
Social services			
Social worker^a^	Annual	34 500.00	Unit Costs of Health and Social Care 2022
Health services			
Doctor (in hospital)^b^	Per hour	145.00	Unit Costs of Health and Social Care 2022
Doctor (not in hospital)^c^	Per consultation	47.00	
Prescription^d^	Per consultation	29.00	
Health services			
Hospital in-patient^e^	Overnight	1404.59	National Schedule of NHS Costs – Year 2021/22
Hospital stay^f^	Short stay	575.91	

NHS, National Health Service.

a. Social worker (adult services), cost per working hour (including qualifications).

b. Consultant psychiatric, cost per working hour (including qualifications).

c. General practitioner, including direct care staff costs (with qualification costs), 9.22 min average per consultation.

d. Prescription costs per consultation, general practitioner (actual cost).

e. Child and Adolescent Mental Health Services, admitted patients.

f. Other mental health disorders, treated by a non-specialist mental health service provider.

#### Intervention costs

The cost of the interventions includes training costs for teachers (where relevant), and delivery costs:

- Training costs consist of costs of instructors – including travel and accommodation where applicable – and training materials such as posters, booklets and associated postage costs. These data were collected directly from the intervention team.
- Data on delivery costs were collected from schools, which reported the amount of time teachers spent on training, preparation and delivery of interventions. It also includes any extra time teachers spent with students as a direct result of the intervention. The teacher’s hourly rates were based on information provided in the finance surveys.

Given the different models for YAM and The Guide, different cost items apply to each intervention. Full details on cost items are included in Table 3. It is assumed that cost per student in the usual school provision group is zero.

**Table 3:** Cost items of interventions

YAM	The Guide
(a) Instructor training costs (b) Printing costs for session materials (c) Dissemination costs for session materials (d) Salary for instructors and helpers (e) Travel and accommodation costs for instructors and helpers (to deliver sessions in schools)	(a) Cost of training school staff to deliver interventions (b) Salary for staff (delivery and preparation time) (c) Printing costs for session materials (d) Dissemination costs for session materials

YAM, Youth Aware of Mental Health programme; The Guide, Mental Health and High School Curriculum Guide.

### Cost-effectiveness analysis

The key research question for this analysis asks whether YAM and The Guide are cost-effective, compared with usual school provision, in terms of HRQoL, at 3–6 months after the start of intervention and at 9–12 months following the end of the intervention. Cost-effectiveness was measured using the incremental cost-effectiveness ratio (ICER), and assessed relative to thresholds typically used by the National Institute for Health and Care Excellence (NICE), which range from £20 000 to £30 000 per QALY gained. Interventions with an ICER below this range are generally considered to be cost-effective.

Cost-effectiveness was conducted from a public health and social care perspective, whereby the costs relating to health and social services, plus school services, were included. Societal costs – such as those resulting from reduced productivity (e.g. parents taking time away from work) – were not included in our analyses because of an absence of data. We also carried out a sensitivity analyses using a public health perspective, in which only costs incurred through use of public health services were included; costs of social services (including those relating to school services) are excluded.

### Statistical analysis

Students that provided responses to HRQoL and CSRI items at baseline and at at least one point of follow-up were included in the analyses. Outcomes relating to HRQoL were estimated with responses to the CHU9D questionnaire. For a QALY score to be calculated, all nine items on the questionnaire must be completed. Missing data – which were assumed to be missing at random – were imputed for responses that completed at least two-thirds of the items. The imputation method is detailed in Deighton et al.^10^ Responses with more than a third of items unavailable were excluded from analyses. A sensitivity analysis was conducted using only responses with complete CHU9D answers.

Differences in costs and outcomes between groups (each intervention against control) was first tested using standard parametric tests. The effect of the intervention on costs and effects were assessed using mixed-effects linear regression models to account for school-level clustering. In addition to the covariates specified in the analysis plan – baseline costs and outcomes, socioeconomic status – we also included the school location and setting, existence of prior interventions and gender as part of the model.

Cost-effectiveness was assessed with the ICER – calculated as the ratio of differences in costs and difference in outcomes. A net-benefit approach was employed, with net benefit calculated as:

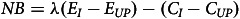

where *NB* is net benefit and *λ* is willingness to pay (WTP) for one unit of improvement in outcomes (e.g. one QALY). *E_I_* and *E_UP_* are the effects of the intervention group and the usual practice group, respectively. *C_I_* and *C_UP_* are the costs of the intervention group and the usual practice group, respectively. WTP values of £20 000 and £30 000 per QALY gained were considered, in line with the NICE guidelines.^20^ Cost-effectiveness acceptability curves were constructed using non-parametric bootstrapping to address uncertainty.

All analyses were run with Stata (version 18 for Windows 10; StataCorp LLC, Texas, USA; https://www.stata.com/).

### Sensitivity analysis

In addition to the complete case analysis, we examined the cost-effectiveness of the interventions from a public health perspective – that is, only costs directly incurred by the public health sector were included – to explore the potential effect of practices in one sector (here, education) on another (health).

## Results

A total of 19 121 year 9 students provided responses for questionnaires at the baseline – 6306 for YAM, 6398 for The Guide and 6417 for usual school provision. At baseline, complete service use data was available for 16 173 students. At the first follow-up, this data was available for 9307 students. For outcome measures, 14 041 students completed the CHU9D at baseline, 8126 students at first follow-up and 10 173 at second follow-up. See Table 4 for data completeness.

**Table 4:** Data completeness (*N* = 19 121)

Number completed	Baseline	Follow-up 1	Follow-up 2
Service use, *n*	16 173	9307	11 697
Child Health Utility 9D Index, *n*			
Completed	14 041	8126	10 173
At least two-thirds completed	15 461	8861	11 184

### Costs

All costs are reported in 2021–2022 values. Total intervention delivery costs for YAM were estimated to be £238 408, with the per student cost at £59. Total cost for The Guide was estimated to be £155 582, which comes to approximately £39 per student. At baseline, health and social care costs were highest for students in schools randomised to The Guide (£1218 for the year before the intervention), and lowest for the usual school provision group (£948). At the first follow-up, the group with the greatest change in service use costs was YAM, with an average increase of £419 per student. This shift is largely attributed to the change in the use of health services, which increased by an average of £318 per student. At the second follow-up, incremental costs for The Guide, relative to usual school provision, was larger than that in the first follow-up, whereas incremental costs for YAM, relative to usual school provision, was smaller than that for the first follow-up. Detailed cost estimates are provided in Table 5.

**Table 5:** Summary of student intervention costs, service use costs by sectors, and student outcomes

Item	YAM	The Guide	Usual school provision
Intervention cost (£)	59.19	38.92	–
Pre-intervention			
Costs (£)			
School services	241.48	223.67	204.79
Social care services	459.12	539.44	423.65
Health services	399.55	430.77	307.70
Hospital (in-patient) services	26.37	23.89	12.20
Total	1126.51	1217.77	948.35
Outcomes (score)			
CHU9D	0.8915	0.8892	0.8938
SMFQ	7.5375	7.4771	7.0284
GHSQ	3.0260	3.0526	2.9942
Follow-up 1			
Costs (£)			
School services	292.96	187.88	233.87
Social care services	433.61	339.63	385.38
Health services	666.93	338.37	513.35
Hospital (in-patient) services	152.27	51.54	46.79
Total	1545.77	917.42	1179.38
Outcomes (score)			
CHU9D	0.9058	0.9022	0.9052
SMFQ	7.5865	7.2132	7.0205
GHSQ	3.0593	3.1151	2.9927
Follow-up 2			
Costs (£)			
School services	212.52	213.71	183.61
Social care services	362.75	342.43	367.00
Health services	409.82	470.24	393.37
Hospital (in-patient) services	26.54	58.52	6.82
Total	1011.63	1084.90	950.80
Outcomes (score)			
CHU9D	0.9229	0.9216	0.9149
SMFQ	7.6249	7.4658	6.8784
GHSQ	3.033	3.0674	3.0674

YAM, Youth Aware of Mental Health programme; The Guide, Mental Health and High School Curriculum Guide; CHU9D, Child Health Utility 9D Index; SMFQ, Short Mood and Feelings Questionnaire; GHSQ, General Help-Seeking Questionnaire.

### Outcomes

At the first follow-up, compared with students in schools randomised to usual school provision, students in schools randomised to The Guide experienced lower QALYs; however, the difference in average QALYs between the two groups was extremely small and not statistically significant. Students in schools randomised to YAM experienced similar outcomes, compared with those in schools randomised to usual school provision. Detailed results for primary outcomes for depressive symptoms and help-seeking behaviour are reported in Deighton et al.^10^ A summary of these results is presented in Tables 6 and 7. At second follow-up, students in schools randomised to either intervention group experienced an improvement in HRQoL outcomes, relative to those in usual school provision. Results of the cost-effectiveness analysis is presented in Table 8 and discussed below.

**Table 6:** Changes in student costs and outcomes (YAM)

Follow-up 1	YAM		Usual practice		Adjusted mean difference		
	*n*	Mean (s.e.)	*n*	Mean (s.e.)	Estimate (s.e.)	95% CI	*p*-value
Cost	2576	486.36 (167.52)	2960	296.14 (110.74)	330.76 (116.35)	102.71−558.81	0.00
Outcomes							
QALYs	4028	0.0143 (0.0021)	4140	0.0114 (0.0020)	−0.0006	−0.0100 to 0.0088	0.90
SMFQ	5118	**−**0.0056 (0.0802)	5914	0.1227 (0.0730)	0.3233 (0.2076)	−0.0834 to 0.7301	0.12
GHSQ	4812	0.0181 (0.0183)	5590	−0.0002 (0.0163)	0.0422 (0.0277)	−0.0120 to 0.0965	0.13

Follow-up 2	YAM		Usual practice		Adjusted mean difference		
	*n*	Mean (s.e.)	*n*	Mean (s.e.)	Estimate (s.e.)	95% CI	*p*-value
Cost	3222	**−**129.03 (94.54)	3993	10.20 (85.33)	118.18	−40.79 to 277.14	0.15
Outcomes							
QALYs	6306	0.0289 (0.0018)	6417	0.0203 (0.0017)	0.0055	−0.0019 to 0.0129	0.14
SMFQ	4600	0.1829 (0.0951)	5424	−0.0139 (0.0830)	0.4182 (0.1960)	0.0341−0.8024	0.03
GHSQ	4336	0.0061 (0.0198)	5120	0.0486 (0.0184)	0.0030 (0.0293)	−0.0545 to 0.0604	0.92

YAM, Youth Aware of Mental Health programme; QALY, quality-adjusted life-years; SMFQ, Short Mood and Feelings Questionnaire; GHSQ, General Help-Seeking Questionnaire.

**Table 7:** Changes in student costs and outcomes (The Guide)

Follow-up 1	The Guide		Usual practice		Adjusted mean difference		
	*n*	Mean (s.e.)	*n*	Mean (s.e.)	Estimate (s.e.)	95% CI	*p*-value
Cost (£)	2801	**−**309.70 (115.61)	2960	296.14 (110.74)	17.10	−184.51 to 218.72	0.87
Outcomes							
QALYs	3997	0.1294 (0.0020)	4141	0.0114 (0.0020)	−0.0025	−0.0109 to 0.0059	0.56
SMFQ	5602	**−**0.1528 (0.7530)	5914	0.1227 (0.0730)	0.1847 (0.2010)	−0.2092 to 0.5786	0.36
GHSQ	5228	0.0756 (0.0186)	5590	**−**0.0002 (0.0163)	0.0867 (0.0345)	0.0192−0.1543	0.01

Follow-up 2	The Guide		Usual practice		Adjusted mean difference		
	*n*	Mean (s.e.)	*n*	Mean (s.e.)	Estimate (s.e.)	95% CI	*p*-value
Cost (£)	3389	**−**168.05 (110.81)	3993	10.20 (85.33)	207.98	40.64−375.32	0.02
Outcomes							
QALYs	6398	0.0288 (0.0017)	6417	0.0203 (0.0017)	0.0044	−0.0031 to 0.0120	0.25
SMFQ	4718	0.2689 (0.0902)	5424	−0.0139 (0.0830)	0.3522	0.0017−0.7228	0.05
GHSQ	4456	0.0058 (0.0207)	5120	0.0486 (0.0184)	0.0310 (0.2956)	−0.0270 to 0.0890	0.30

The Guide, Mental Health and High School Curriculum Guide; QALY, quality-adjusted life-years; SMFQ, Short Mood and Feelings Questionnaire; GHSQ, General Help-Seeking Questionnaire.

**Table 8:** Cost-effectiveness of interventions, public health and social care perspective (with imputed Child Health Utility 9D Index data)

Follow-up 1	Mean difference	
	YAM versus usual practice	The Guide versus usual practice
Incremental costs (£)	330.76	17.10
Incremental effects (QALYs)	−0.0006	−0.0025
Incremental cost-effectiveness ratio (£ per QALY gained)	Usual practice dominant	Usual practice dominant

Follow-up 2		
Incremental costs (£)	118.18	207.98
Incremental effects (QALYs)	0.0055	0.0044
Incremental cost-effectiveness ratio (£ per QALY gained)	21 503	47 078

YAM, Youth Aware of Mental Health programme; The Guide, Mental Health and High School Curriculum Guide; QALY, quality-adjusted life-years.

### Cost-effectiveness

Results from the first follow-up suggests that, relative to the students at schools randomised to usual school provision, those who attended sessions of YAM and The Guide experienced poorer outcomes in terms of QALY. However, it should be noted these differences are only marginal (**−**0.0006 QALYs and **−**0.0025 QALYs, respectively). Costs for both intervention arms were higher than that for usual school provision. Based on the cost-effectiveness thresholds set by the NICE of between £20 000 and £30 000 per QALY gained, neither intervention would be considered cost-effective. Results from bootstrapping indicate that at the £30 000 WTP threshold, YAM has a 4% probability of being considered cost-effective; for The Guide, this is 31%.

Results at the second follow-up show that, relative to students at schools randomised to usual school provision, students in schools randomised to YAM and The Guide experienced marginally higher HRQoL outcomes (0.0055 QALYs and 0.0044 QALYs, respectively). Costs for both intervention arms were still higher than that for usual school provision. For YAM, using results at second follow-up gives an ICER of £21 503 per QALY gained. For The Guide, the ICER at second follow-up was £47 078.

Results from the two sensitivity analyses are presented in Tables 9 and 10. We note that in the case of a strictly public health perspective, both interventions are potentially considered cost-effective at second follow-up. However, as this does not take into account costs incurred in school or social services, this is likely an underestimate of the overall costs. In all other scenarios, the interventions are not considered to be cost-effective.

**Table 9:** Cost-effectiveness of interventions, public and social care perspective (with complete Child Health Utility 9D Index responses)

Follow-up 1	Mean difference	
	YAM versus usual practice	The Guide versus usual practice
Incremental costs	446.50	95.69
Incremental effects (QALYs)	−0.0127	−0.0080
Incremental cost-effectiveness ratio (£ per QALY gained)	Usual practice dominant	Usual practice dominant

Follow-up 2		
Incremental costs	12.70	170.83
Incremental effects (QALYs)	−0.0071	−0.0047
Incremental cost-effectiveness ratio (£ per QALY gained)	Usual practice dominant	Usual practice dominant

YAM, Youth Aware of Mental Health programme; The Guide, Mental Health and High School Curriculum Guide; QALY, quality-adjusted life-years.

**Table 10:** Cost-effectiveness of interventions, public health perspective (with imputed Child Health Utility 9D Index data)

Follow-up 1	Mean difference	
	YAM versus usual practice	The Guide versus usual practice
Incremental costs	179.04	−0.08
Incremental effects (QALYs)	−0.0006	−0.0025
Incremental cost-effectiveness ratio (£ per QALY gained)	Usual practice dominant	Not applicable

Follow-up 2	Mean difference (95% CI)	
Incremental costs	41.29	87.38
Incremental effects (QALYs)	0.0055	0.0044
Incremental cost-effectiveness ratio (£ per QALY gained)	7504	19 779

YAM, Youth Aware of Mental Health programme; The Guide, Mental Health and High School Curriculum Guide; QALY, quality-adjusted life-years.

## Discussion

This study contributes to the body of literature in terms of the health economic analysis of universal school-based interventions in addressing student emotional health and well-being. Our analysis found that neither the YAM programme nor The Guide demonstrated cost-effectiveness at the first follow-up, with marginal differences in both costs and outcomes compared with usual practice. At the second follow-up, the YAM intervention showed a higher probability of being cost-effective relative to usual practice, although this was based on small improvements in outcomes. These findings are consistent with previous research, which has similarly highlighted the challenges of demonstrating the economic value of universal mental health interventions in schools.^5^ Although school-based approaches may have potential, our results suggest that the benefits of such interventions may be more pronounced in the medium to long term, with mechanisms such as increased mental health literacy and peer support networks contributing to cost-savings.

This is one of the largest trials on a range of school-based preventative interventions in England, providing robust data that reflects a wide range of school settings, increasing the external validity of the results. This study adds to the sparse literature on economic evaluations of school-based mental health interventions, assessing not only the costs of implementation, but also changes in service use costs associated with mental health and well-being resulting from the interventions. Detailed data on delivery costs and resources used were collected directly from participants by the research team, which improves understanding of the requirements for implementing such interventions.

Results from the health economic analysis of YAM and The Guide suggest that, based on responses from the first follow-up, there is a low probability that either intervention would be considered cost-effective. There was no evidence to suggest that students in schools randomised to either of the intervention arms had superior outcomes to those in the control arm. Therefore, there is a very low probability that either intervention would be considered cost-effective in the short run. These findings are consistent with those from existing studies of similar programmes, as found by reviews such as that of Mackenzie and Williams^4^ – in that effectiveness of school-based universal interventions were neutral or small, with mixed results regarding efficacy – and trials such as MY Resilience In ADolescence.^3^ Results from the second follow-up, based on responses from 9 to 12 months after the end of intervention(s), indicate a higher probability (>50%) of YAM being considered cost-effective compared with usual practice. Although service use costs of the intervention group were still higher than that of the usual school provision group, this difference is notably smaller at the second follow-up (£118) compared with the first follow-up (£330).

The specific mechanisms underlying these changes are not entirely clear given the available data. Although cost-savings appear to result primarily from reduced use of health services, it remains uncertain whether this reduction is directly attributable to improved emotional wellbeing. Other potential mechanisms might include increased mental health literacy and awareness, which could lead to earlier self-management and reduced reliance on formal services, or enhanced problem-solving skills fostered by YAM, enabling students to cope more effectively with stressors without needing external support.^21^ Additionally, the group setting of the intervention may have contributed to peer support networks, further reducing the need for formal health services.^22^ This raises important questions regarding the general applicability of these programmes, particularly in diverse educational settings. It also raises the question of whether programmes of this design should be implemented, or whether alternative measures – such as targeted interventions for vulnerable populations, with associated screening processes – might be a better use of resources.

This study has its limitations. Self-reported data, particularly on service use and quality of life, may be subject to recall bias and/or social desirability bias, which may influence the precision of cost estimates. Studies have found that self-reported service use can be overestimated, when compared with administrative records.^23^ Additionally, we do not have certainty on whether students in this age group will reliably track and report their service use. However, any bias is likely to be consistent across both the intervention and control groups, suggesting that any impact on relative comparisons or cost-effectiveness estimates should be minimal.

We note also there is attrition in responses at both points of follow-up, relative to the baseline. Future studies on such interventions may benefit from more in-depth investigation as to whether the likelihood of being lost to follow-up is related to student characteristics (e.g. free school meal status). Similarly, subgroup analyses (e.g. by gender) may provide insight as to the type of students who are more likely to benefit from these interventions. In both intervention arms, more responses were received from female students than male students, whereas the opposite was true for the usual practice arm. The reason for this imbalance was outside the scope of the trial, and may be a potential topic for further investigation.

There is also a degree of uncertainty in the reports from staff involved in the delivery of interventions, and the categorical nature of response options (e.g. ‘preparation time’) may not fully capture the time spent, leading to potential inaccuracies. However, these types of responses are less cognitively taxing,^24^ and this limitation is difficult to avoid without time-tracking data, which would not be feasible. Furthermore, we utilised national average values when estimating resource use costs. Although this approach is standard practice in economic evaluations, it may not fully account for geographic or setting-specific cost variations. The exclusion of broader societal costs, such as productivity losses for parents, may have led to an underestimation of the full economic impact of these interventions.

As well as this, the follow-up period of up to a year following the end of the intervention may limit our ability to capture potential longer-term effects of the intervention. Although the two follow-ups provide early insights, they may not fully capture the long-term benefits of the interventions, particularly in terms of sustained mental health improvements and reduced service use. Longer-term outcomes, especially those related to educational attainment and adult mental health, remain uncertain. Although this trial has a relatively long time horizon compared with similar studies,^25^ there is still a need for longer-term follow-ups on both costs and outcomes of participants, as evidence suggests that the full benefits of universal preventive mental health interventions may emerge over a longer time horizon,^26^ Longer-term follow-up would also provide a clearer understanding of how these interventions influence educational attainment and adult mental health outcomes, which are important for assessing the interventions’ full cost-effectiveness.

In conclusion, the AWARE trial provides important insights into the cost-effectiveness of school-based mental health interventions for year 9 students in England. Findings are in line with previous literature suggesting that school-based universal interventions have limited effects on students in terms of QALYs. Although there is some potential for YAM to be cost-effective in the medium term, these calculations are dependent on small changes in outcomes. Our findings are in line with previous analyses of interventions of similar design. Future efforts of improving student mental health and well-being may benefit from increased focus on individual-level, targeted interventions that address the needs of high-risk groups. Additionally, longer follow-up periods are necessary to fully capture the long-term impacts of these interventions, particularly regarding educational attainment and adult mental health outcomes.

## Data Availability

An anonymised data-set will be made publicly available in the future (planned for 2026).
